# Binding of amelogenin to MMP-9 and their co-expression in developing mouse teeth

**DOI:** 10.1007/s10735-012-9423-1

**Published:** 2012-05-31

**Authors:** Junsheng Feng, Jennifer S. McDaniel, Hui-Hsiu Chuang, Ouwen Huang, Audrey Rakian, Xiaoping Xu, Bjorn Steffensen, Kevin J. Donly, Mary MacDougall, Shuo Chen

**Affiliations:** 1 Department of Developmental Dentistry, The University of Texas Health Science Center at San Antonio, 7703 Floyd Curl Dr., San Antonio, TX 78229-3900 USA; 2 Department of Periodontics, The University of Texas Health Science Center at San Antonio, San Antonio, TX 78229 USA; 3 Department of Anatomy and Histoembryology, Fujian Medical University, Fuzhou, 350018 China; 4 Department of Oral/Maxillofacial Surgery, School of Dentistry, University of Alabama at Birmingham, Birmingham, AL 35294 USA

**Keywords:** Amelogenin, MMP-9, Co-expression, Protein interaction, Ameloblasts

## Abstract

Amelogenin is the most abundant matrix protein in enamel. Proper amelogenin processing by proteinases is necessary for its biological functions during amelogenesis. Matrix metalloproteinase 9 (MMP-9) is responsible for the turnover of matrix components. The relationship between MMP-9 and amelogenin during tooth development remains unknown. We tested the hypothesis that MMP-9 binds to amelogenin and they are co-expressed in ameloblasts during amelogenesis. We evaluated the distribution of both proteins in the mouse teeth using immunohistochemistry and confocal microscopy. At postnatal day 2, the spatial distribution of amelogenin and MMP-9 was co-localized in preameloblasts, secretory ameloblasts, enamel matrix and odontoblasts. At the late stages of mouse tooth development, expression patterns of amelogenin and MMP-9 were similar to that seen in postnatal day 2. Their co-expression was further confirmed by RT-PCR, Western blot and enzymatic zymography analyses in enamel organ epithelial and odontoblast-like cells. Immunoprecipitation assay revealed that MMP-9 binds to amelogenin. The MMP-9 cleavage sites in amelogenin proteins across species were found using bio-informative software program. Analyses of these data suggest that MMP-9 may be involved in controlling amelogenin processing and enamel formation.

## Introduction

Dental enamel is formed by ameloblasts originally derived from embryonic oral epithelium. The differentiation of dental epithelium into functional ameloblasts occurs in spatial–temporal patterns during tooth development and these ameloblasts synthesize and secrete enamel matrix proteins. Amelogenin is the most abundant enamel matrix protein, accounting for about 90 % of total enamel organic matrix. This gene was cloned and characterized from the teeth of bovine, porcine, rat, mouse and human (Snead et al. 1983; Takagi et al. 1984; Gibson et al. 1991a; Nakahori et al. 1991; Salido et al. 1992; Bonass et al. 1994; Hu et al. 1996; Diekwisch et al. 1997). It is synthesized in a distinct time frame during amelogenesis. In mice, amelogenin gene expression was identified in ameloblasts and enamel matrix from embryonic day 15 to postnatal day 14 (Snead et al. 1988; Couwenhoven and Snead 1994; Hu et al. 2001; Iacob and Veis 2006). Rat amelogenin expression was also characterized in teeth from embryonic day 18.5 to postnatal day 15 as well as the continuously erupting incisors at the later stages (Fong et al. 1996; Inage et al. 1996; Bleicher et al. 1999). In developing hamster teeth, amelogenin expression was identified in pre-ameloblasts, secretory, transition, and early maturation stage ameloblasts and in the enamel matrix (Karg et al. 1997). A similar expression pattern was observed in pig teeth except for ameloblasts after the transition stage (Wakida et al. 1999). Originally, amelogenin was thought to be expressed solely by ameloblasts during tooth development (Snead et al. 1988; Inai et al. 1991; Karg et al. 1997; Hu et al. 2001). Recently, it has been found that this gene is also expressed in odontoblasts and other tissues (Veis et al. 2000; Oida et al. 2002; Nagano et al. 2003; Papagerakis et al. 2003; Iacob and Veis 2006; Haze et al. 2007). Amelogenin plays a critical role in the structural organization of enamel as well as in mineralization (Deutsch et al. 1995; Bartlett and Simmer 1999). Amelogenin gene mutations in humans and mice cause amelogenesis imperfecta (AI), one of the most common enamel genetic diseases (Lagerström et al. 1991; Aldred et al. 1992; Gibson et al. 2001; Wright et al. 2011).

Large-molecular-mass amelogenin proteins as well as small-molecular-mass amelogenin polypeptides have been isolated and characterized in different species teeth during the process of development and mineralization (Bronckers et al. 1995; Boabaid et al. 2004). The biochemistry and biological roles of these different fragments in enamel formation and biomineralization have been studied (Gibson et al. 1991b; Le et al. 2007; Warotayanont et al. 2008; Nakayama et al. 2010; Pugach et al. 2010). It is known that amelogenin requires processing by proteinases to activate functional domains (Fincham and Moradian-Oldak 1993). For instance, matrix metalloproteinase 20 (MMP-20) and kallikren 4 (Klk4) are expressed in ameloblasts during amelogenesis and are capable of catalyzing enamel matrix proteins including amelogenin (Li et al. 1999; Ryu et al. 1999; Bourd-Boittin et al. 2004; Sun et al. 2010; Uskoković et al. 2011). These two proteinases are critical for enamel matrix processing. MMP-20 and Klk4 gene mutations in humans and mice cause hypomaturation AI (Caterina et al. 2002; Hart et al. 2004; Kim et al. 2005; Simmer et al. 2009).

Matrix metalloproteinase 9 (MMP-9), also known as gelatinase B or type IV collagenase, belongs to a member of the MMP gene family and is expressed in ameloblasts and odontoblasts as well as other dental cells during tooth development (Tjäderhane et al. 1998; Sahlberg et al. 1999; Randall and Hall 2002; Goldberg et al. 2003; Palosaari et al. 2003; Yoshiba et al. 2003; Takahashi et al. 2006; Paiva et al. 2009; Gomes et al. 2011). This enzyme is involved in bone resorption and degradation of the basement membrane during tooth development as well as extracellular matrix (ECM) turnover in association with tooth eruption (Linsuwanont et al. 2002; Basi et al. 2011). MMP-9 has a broad range of substrate specificity including native collagenous and non-collagenous proteins as well as non-structural ECM components (Vu et al. 1998; Kridel et al. 2001; Somerville et al. 2003; Nagase et al. 2006). However, the entire assortment of substrates for the MMPs has not been fully elucidated. MMP inhibitors were used to treat mouse tooth germs, resulting in impairment of murine amelogenesis (Fanchon et al. 2004; Bourd-Boittin et al. 2005). Furthermore, bone deficiency was observed and osteoblast apopotosis was increased in MMP-9 knock out mice (Vu et al. 1998). Our previous study showed that abnormal tooth morphology, immature ameloblast differentiation, and loss of ameloblast polarization occur in MMP-9 null mice (Yuan et al. 2009, unpublished data). Although expression of both amelogenin and MMP-9 during tooth development was described, their relationship in tooth formation has not been observed. In the present study, we were interested in the interaction between amelogenin and MMP-9 as well as comparing the spatial–temporal distribution of both proteins during mouse tooth development. We hypothesized that interactions between amelogenin and MMP-9 as well as developmental changes in the spatial distribution of these two proteins might exist. To test these hypotheses, we designed experiments to examine the binding of MMP-9 to amelogenin. We further analyzed the distribution of amelogenin and MMP-9 at the same stages of mouse tooth development. Our studies indicated that MMP-9 binds to amelogenin and they are co-expressed in ameloblasts in developing mouse teeth. Furthermore, computational data analysis indicated that potential cleavage sites of MMP-9 exist in amelogenin across different species.

## Materials and methods

### Animals and tissue preparation

All experimental procedures involving the use of animals were approved by the University of Texas Health Science Center at San Antonio (UTHSCSA), TX. ICR mice were purchased from Harlan-Laboratory Animals Inc. (Indianapolis, IN, USA). The MMP-9 knock out mice were obtained from the Jackson Laboratory (Bar Harbor, Maine, USA). For developmental studies, mice with litters of embryonic stage E18.5 and postnatal days 2, 5 and 7 were sacrificed. Mouse tissues were dissected and fixed in 4 % paraformaldehyde overnight. After demineralization in 15 % EDTA, samples were dehydrated in increasing concentrations of ethanol, embedded in paraffin, sectioned, and prepared for immunohistochemistry analysis.

### Cell culture

Mouse odontoblast-like cells (MO6-G3) were grown at 33 °C under 5 % CO_2_ in alpha minimum essential medium (α-MEM) supplemented with 10 % fetal calf serum, 100 units/ml penicillin/streptomycin, 50 μg/ml ascorbic acid, and 10 mM Na β-glycerophosphate (Sigma, St. Louis, MO, USA). The mouse enamel organ epithelial (EOE-3M) cell line was maintained in Dulbecco’s modified eagle medium (DMEM) (Gibco, Grand Island, NY, USA) with 10 % fetal calf serum, 100 units/ml penicillin/streptomycin, 50 μg/ml ascorbic acid.

### RNA preparation and reverse transcription-polymerase chain reaction (RT-PCR)

Total RNA was extracted from mouse teeth and MO6-G3 and EOE-3M cell lines by using RNA STAT-60 kit (Tel-Test, Inc. Friendswood, TX, USA), treated with DNase I (Promega, Madison, WI, USA), and purified with the RNeasy Mini Kit (Qiagen Inc., Valencia, CA, USA). RNA concentration was determined at an optical density of OD_260_. The RNA was transcribed into cDNA by SuperScript II reverse transcriptase (Invitrogen, Carlsbad, CA, USA). For PCR analysis, specific primers of mouse amelogenin and MMP-9 were synthesized as follows: mouse amelogenin, forward 5′-TGAAGTGGTACCAGAGCA-3′ and reverse 5′-ACAGGGATGATTTGGTGGTG-3′; MMP-9, forward 5′-CAGACCAAGAGGGTTTTCTT-3′ and reverse CTTGTTCACCTCATTTTGGA-3′. The PCR reaction was first denatured at 95 °C for 5 min, and then carried out at 95 °C for 30 s, at 55–60 °C for 30 s and at 72^o^ C for 60 s for 35 cycles and with a final 10 min extension at 72 °C. Five μl of PCR products were analyzed using agarose gels and ethidium bromide staining. Corrective DNA was verified by DNA sequencing.

### Expression and purification of recombinant proteins

The full-length mouse amelogenin gene was amplified by PCR using mouse tooth cDNA as a template with primers adding *Xho* I and *Not* I sites for directional ligation into the expression vector pGEX-6P1 with *Xho* I and *Not* I sites (Amersham Pharmacia Biotech, Piscataway, NJ, USA) and named pGST196 (amino acids^1–196^). The pGST196 expression and purification was performed according to the manufacturer’s instruction (Amersham Pharmacia Biotech). The recombinant, untagged murine amelogenin (rM179, amino acids^18–196^) was subcloned into pET11a vector with *Nde*l and *BamH* I sites (Novagen, Madison, WI, USA) and termed pET179. This pET179 vector was kindly provided by Dr. Simmer (The Department of Biological and Materials Sciences, University of Michigan School of Dentistry, Ann Arbor, MI, USA). A recombinant amelogenin protein was expressed and purified as described previously (Simmer et al. 1994). Recombinant mutant MMP-9 (rMut-MMP-9) construct was generated as described previously (Xu et al. 2005). Briefly, to obtain rMut-MMP-9 with intact ligand-binding properties but without catalytic activities, the active site Glu^402^ of proMMP-9 was substituted for alanine residue to generate MMP-9^E402A^ (Morgunova et al. 1999). The point mutation was introduced into the coding DNA by overlap-extension PCR using the following primer pairs: forward, 5′-GTGGCGGCGCATGCGTTCGGCCACGCG-3′, and reverse, 5′-CGCGTGGCCGAACGCATGCGCCGCCAC-3′. The expression construct for MMP-9 in pRSETA vector served as a template in PCR reaction buffer that includes 5 μl of 10× reaction buffer, 100 ng of template DNA, 125 ng of each primer, 25 μM dNTP mixtures, and 2.5 units Pfu Turbo DNA polymerase. The cycles included 95 °C for 2 min, then 95 °C for 30 s, 55 °C for 1 min and 72 °C for 10 min for 12 cycles. Subsequently, 1 μl of the DpnI restriction enzyme was added for 1 h at 37 °C to digest the parental DNA. The pRESTA vector containing histidine tagged rMut-MMP-9 gene was transformed into *E. coli* BL21 (DE3) competent cells and expressed and purified. A recombinant mouse MMP-9 protein was purchased from R&D Systems Inc. (Minneapolis, MN, USA; Catalog No. 909-MM).

### Glutathione fusion protein (GST) pull down assay

For probing protein–protein interactions, either the full length recombinant amelogenin protein tagged with GST (pGST196) or GST protein was incubated with the recombinant mutant MMP-9 protein in the lysis buffer (20 mM Tris–HCl, pH 8.0, 200 mM NaCl, 1 mM EDTA) overnight at 4 °C with end–over-end mixing. After the reaction, the glutathione agarose beads were added for further incubation. The samples were then centrifuged and the supernatant was removed. After extensive washes, the beads were mixed with an equal volume of 2× SDS-PAGE gel-loading buffer and then boiled for 4 min followed by SDS-PAGE and Western Blot analyses.

### Western blot analysis

The proteins were loaded onto a 10 % SDS-PAGE gel and transferred to a trans-blot membrane (Bio-Rad Laboratory, Inc., Hercules, CA, USA). Western blotting assay was carried out as described earlier (Chen et al. 2008).

### Gelatin zymography

Gelatinase activity of MO6-G3 and EOE-3M cells was determined by SDS-PAGE electrophoresis zymography. The supernatant of MO6-G3 and EOE-3M without serum treatment underwent electrophoresis without reduction on SDS-PAGE gels prepared with 7 % acrylamide containing 0.1 % gelatin. The SDS was removed by a 1-h incubation in 2.5 % Triton X-100, and the gels were then incubated in 30 mM Tris–HCl (pH 7.4), 200 mM NaCl, 5 mM CaCl_2_, and 1 mM ZnCl_2_ at 37 °C for overnight prior to being stained with Coomassie Brilliant Blue. Enzyme activity was visualized as zones of gelatin clearance.

### Immunohistochemistry

Primary antibodies including a goat polyclonal anti-Amel (C-19), a rabbit polyclonal anti-Amel (FL-191), a goat polyclonal anti-MMP-9 (C-20) and a rabbit polyclonal anti-MMP-9 (H-129) were purchased from Santa Cruz Biotechnology, Inc. (Santa Cruz, CA, USA). The IHC experiments were performed using an ABC kit (Vector Laboratories Inc. Burlingame, CA, USA), according to the manufacturer’s instructions. Either pre-immune rabbit or goat IgG instead of the first antibodies was used as a negative control (Dakocytomation, Carpinteria, CA, USA). For observation of co-expression of amelogenin and MMP-9, the double-labeled immunostaining was carried out using two types of primary antibodies along with two types of fluorescent secondary antibodies. The tissue section was blocked with normal donkey serum (Sigma, St. Louis, MO, USA) for 60 min at room temperature and then incubated with one type of the primary goat polyclonal anti-amelogenin antibody, C-19, which was recognized by the donkey anti-goat secondary antibody conjugated with Alexa Fluo^®^ 488 (Molecular Probes, Eugene, OR, USA) followed by incubation with a rabbit polyclonal antibody against MMP-9, H-129, recognized by the donkey anti-rabbit secondary antibody conjugated with Alexa Fluo^®^ 568. The immunostained section using the two types of antibodies was observed under the same parameters in a Nikon inverted microscope and quantitated by means of NIS-GIEMENTS software. For each experiment, all slides were simultaneously processed for a specific antibody, so that homogeneity in the staining procedure was ensured between the samples. After the capture of these images at the same magnification, the threshold was set and maintained for each slide in the experiment. The optical density was calculated by use of the morphometric analysis within the software package. Hoechst was used for nucleus staining and either normal rabbit or goat immunoglobulin (IgG) served as a negative control (Dakocytomation).

### Confocal microscopic evaluation and image acquisition

Sections were evaluated with a Nikon Eclipse 90i C1si laser scanning confocal microscope (Nikon Instruments, Melville, NY, USA) with a 40X/1.30 N.A. oil immersion objective. This evaluation included the selection of a laser gain setting used to image Na_v_1.6-immunofluorescence to avoid saturated pixels so to allow a full dynamic range of Na_v_1.6-immunofluorescence pixel intensity and to select laser gain settings used to image caspr staining.

### Protein sequencing and data analyses

A database search was performed at the National Center for Biotechnology Information website (http://www.ncbi.nlm.nih.gov/blast) using the BLAST program. The amelogenin nucleotides and derived amino acids were aligned with those from different species using the Gene Runner software program (http://www.generunner).

## Results

### Expression of amelogenin and MMP-9 in mouse teeth

**Fig. 1:**
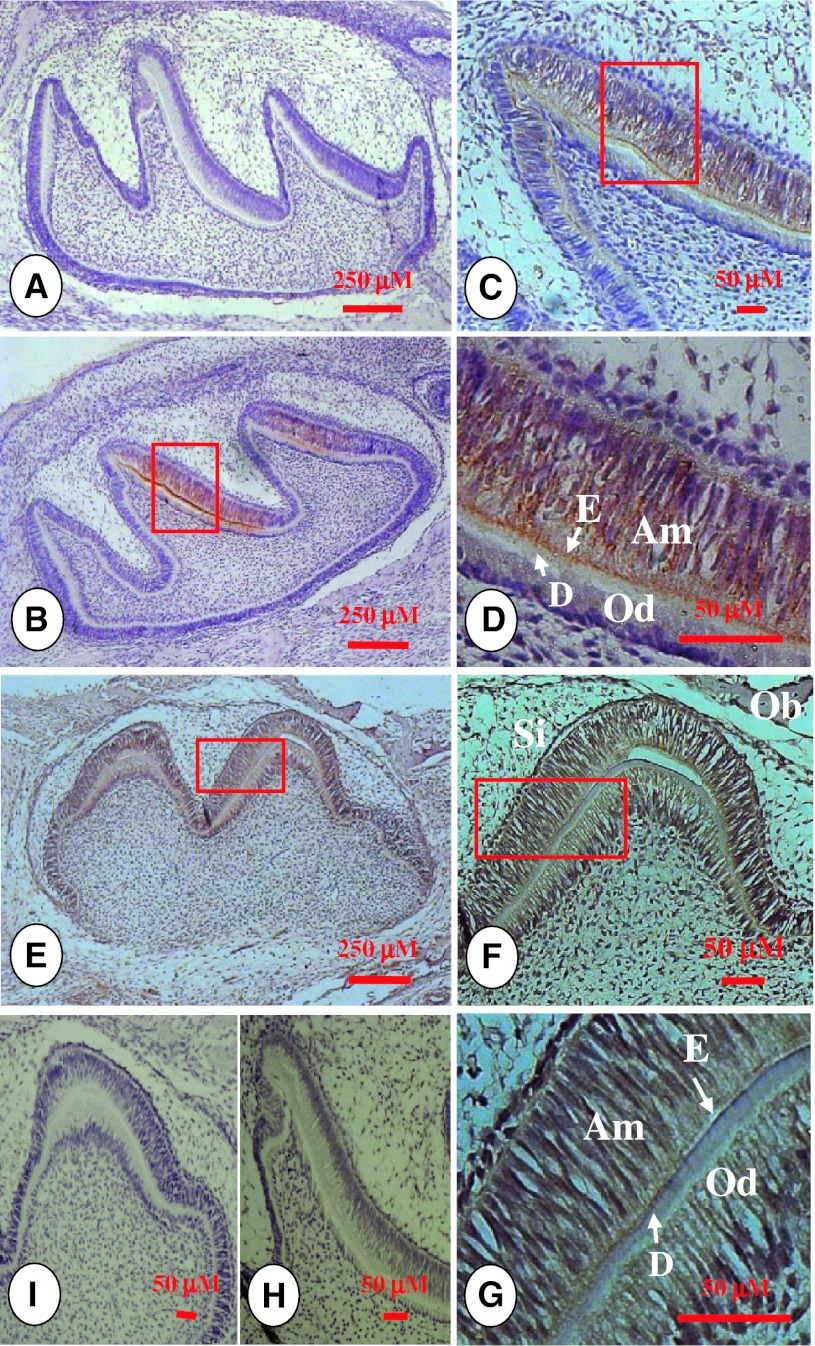
Amelogenin and MMP-9 expression patterns at postnatal day 2 in developing teeth. **b** Amelogenin expression was observed in pre-ameloblasts, ameloblasts, enamel matrix and odontoblasts at postnatal day 2 in mouse molars. **c**, **d** were higher magnification of the *boxed area* in **b**. **a** Pre-immune IgG at the same concentration as anti-amelogenin antibody was used as a negative control. **e** Mouse molar was stained with anti-MMP-9 antibody. **f**, **g** show higher magnification of the *boxed area* in **e**. MMP-9 was widely expressed in bone mesenchymal cells, stratum intermedium, dental pulp, odontoblasts, ameloblasts and enamel matrix. **i** The section was stained with pre-immune IgG as a negative control. **h** A tissue section from a MMP-9 knock out mouse was incubated with anti-MMP-9 antibody as control. *Am* ameloblasts, *D* dentin, *E* enamel, *Od* odontoblasts, *Ob* bone mesenchymal cells, *Si* stratum intermedium

**Fig. 2:**
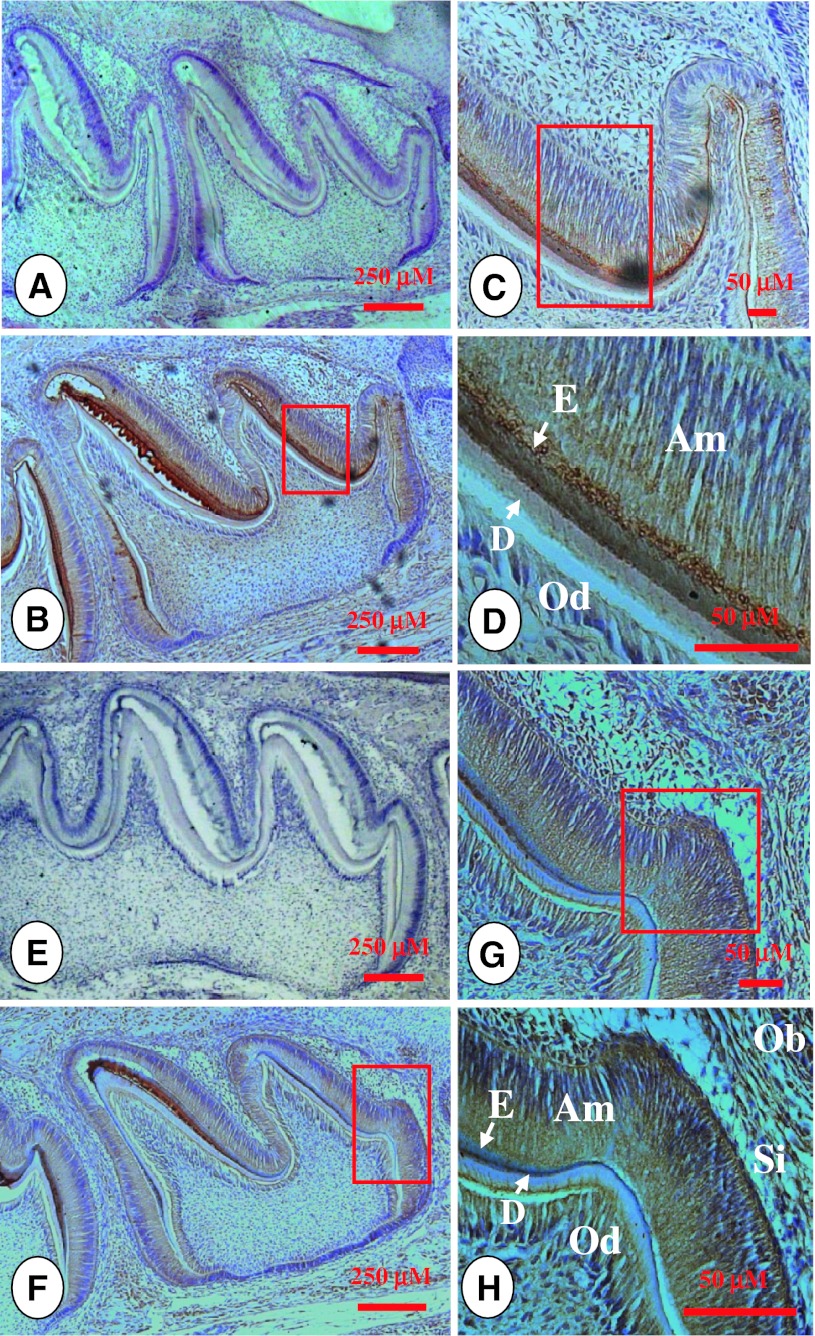
Amelogenin and MMP-9 expression patterns at postnatal day 5 in developing teeth. **b** Expression of amelogenin protein was found in ameloblasts, enamel matrix and odontoblasts. **c**, **d** were higher magnification of the *boxed area* in **b**. **a** Pre-immune IgG at the same concentration as the anti-amelogenin antibody was used as a negative control. **f** Immunostaining indicated that MMP-9 was widely expressed in ameloblasts, odontoblasts and enamel matrix and other cells. **g**, **h** showed higher magnification of the *boxed area* in **f**. **e** Tissue section was incubated with pre-immune IgG as control. *Am* ameloblasts, *D* dentin, *E* enamel, *Od* odontoblasts, *Ob* bone mesenchymal cells, *Si* stratum intermedium

**Fig. 3:**
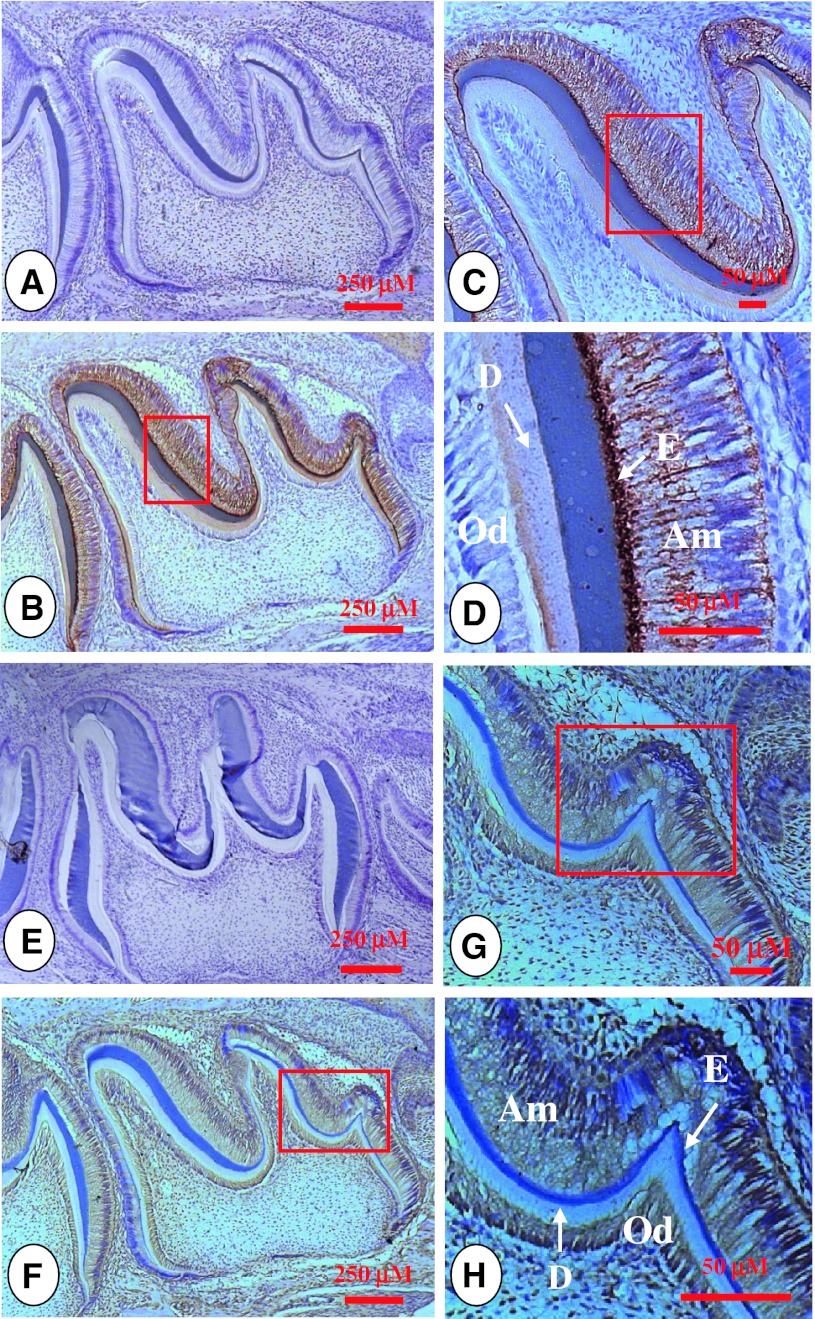
Amelogenin and MMP-9 expression patterns at postnatal day 7 in developing teeth. Both amelogenin and MMP-9 were expressed in ameloblasts, enamel matrix and odontoblasts. **b**. Tissue section was stained with anti-amelogenin antibody. **c**, **d** showed higher magnification of the *boxed area* in **b**. Expression of amelogenin was detected in ameloblasts and the enamel matrix. Also, low levels of amelogenin were found in odontoblasts. **a** Negative control. **f** Immunostaining indicated that MMP-9 was widely expressed in other cells besides ameloblasts, odontoblasts and the enamel matrix. **g**, **h** showed higher magnification of the *boxed area* in **f**. **e** Tissue section was incubated with pre-immune IgG as a negative control. *Am* ameloblasts, *D* dentin, *E* enamel, *Od* odontoblasts

To determine the expression of the two genes in ameloblasts, we systematically evaluated the expression of amelogenin and MMP-9 in developing mouse teeth using immunohistochemistry assays. Amelogenin epitopes were highly localized in the pre-ameloblasts, secretory ameloblasts and enamel matrix at the postnatal day 2 (PN2) in mouse molars (Fig. 1b–d). MMP-9 expression overlapped with amelogenin in these regions and its signal was also present in the dental pulp cells, alveolar bone mesenchymal cells and stratum intermedium (Fig. 1e–g). At PN 5, amelogenin protein in secretory ameloblasts and enamel layer was strongly expressed, but low levels of expression were observed in odontoblasts and stratum intermedium (Fig. 2b–d). The spatial distribution pattern of MMP-9 was similar to amelogenin besides its expression in alveolar bone mesenchyme (Fig. 2f–h). Similar to PN 5, at PN7, amelogenin was highly expressed in secretory ameloblasts and enamel in developing molars, whereas its signal was weakly detected in odontoblasts (Fig. 3b–d). Compared to amelogenin, the MMP-9 gene was widely expressed although MMP-9 was also present in ameloblasts and odontoblasts (Fig. 3f–h).

**Fig. 4:**
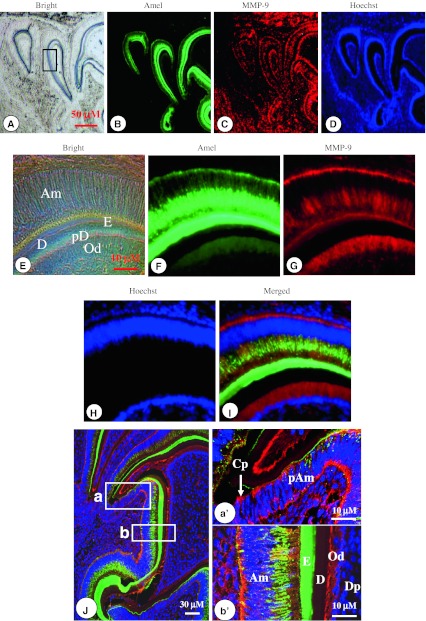
Co-expression of amelogenin and MMP-9 in developing teeth. Amelogenin expression was detected in pre-ameloblasts, ameloblasts, enamel matrix and odontoblasts. However, the highest level of expression was in secretory ameloblasts and the enamel layer (**b**, **f**, **i**). Amelogenin protein was mostly located within the supra-nuclear area (towards the enamel) in secretory ameloblasts. MMP-9 was also expressed in these areas overlapped with amelogenin (**c**, **g**, **i**). **d**, **h** The cells were stained with Hoechst for the nucleus. **i** Image was merged. *Bar* = 10 μM. **j** Confocal micrographs of collapsed z-projection images (consisting of 20 z-sections with spacing increments of 1 mM) show amelogenin (*green color*) and MMP-9 (*red color*) staining relationships within ameloblastic cells and enamel matrix of a tooth section from a postnatal day 4. **a′**, **b′**. Higher magnification of the *boxed area* in **j**-**a**, **b**: amelogenin expression was weakly detected in pre-ameloblasts at the cervical loop (Cp) region, but MMP-9 expression (*red color*) was seen within the cytoplasm in pre-ameloblasts. Co-expression of amelogenin and MMP-9 proteins was visible within the cytoplasm of ameloblasts and in the enamel layer. High expression of amelogenin was observed in secretory ameloblasts (Am) and enamel matrix (E)

To further study the co-expression of the two proteins in ameloblasts, their distribution profiles in developing mouse teeth were examined using double labeling immunohistochemistry. Amelogenin protein in secretory ameloblasts was strongly expressed, and the signal intensity per cell was higher in enamel matrix (Fig. 4b, f). This protein in secretory ameloblasts was mostly located in the supranuclear area (towards the enamel), but was also present in the infranuclear region (away from the enamel). Amelogenin expression was also detected in odontoblasts, but its expression level in odontoblasts was much lower than ameloblasts and the enamel layer. MMP-9 signal coincided with the expression of amelogenin in ameloblasts, enamel layer as well as odontoblasts (Fig. 4c, g). However, the MMP-9 expression pattern was widely detected in other tissues besides ameloblasts, enamel matrix and odontoblasts. To further confirm their co-localization, tissue sections from PN4 were examined using the confocal microscope. The data showed that their co-expression was seen mostly within the cytoplasm of the ameloblasts (Fig. 4j).

### Expression of amelogenin and MMP-9 in mouse enamel organ epithelial and odontoblast-like cells

**Fig. 5:**
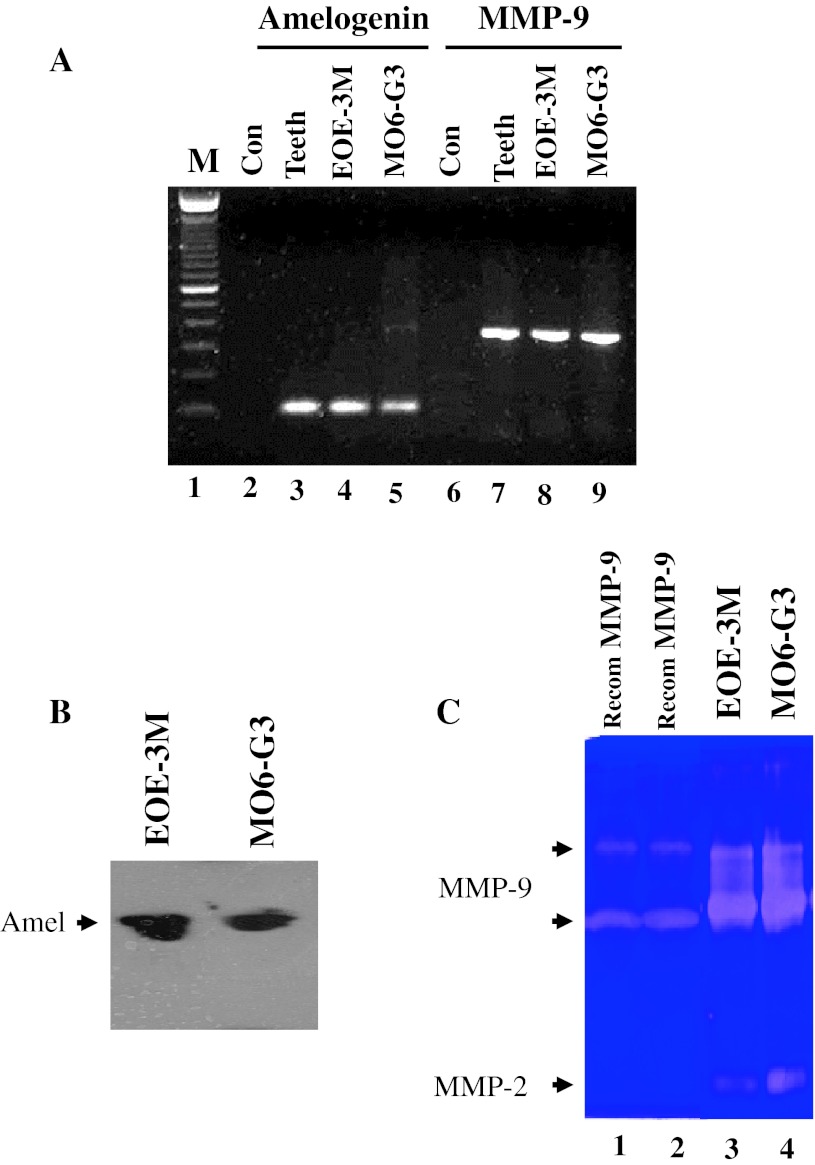

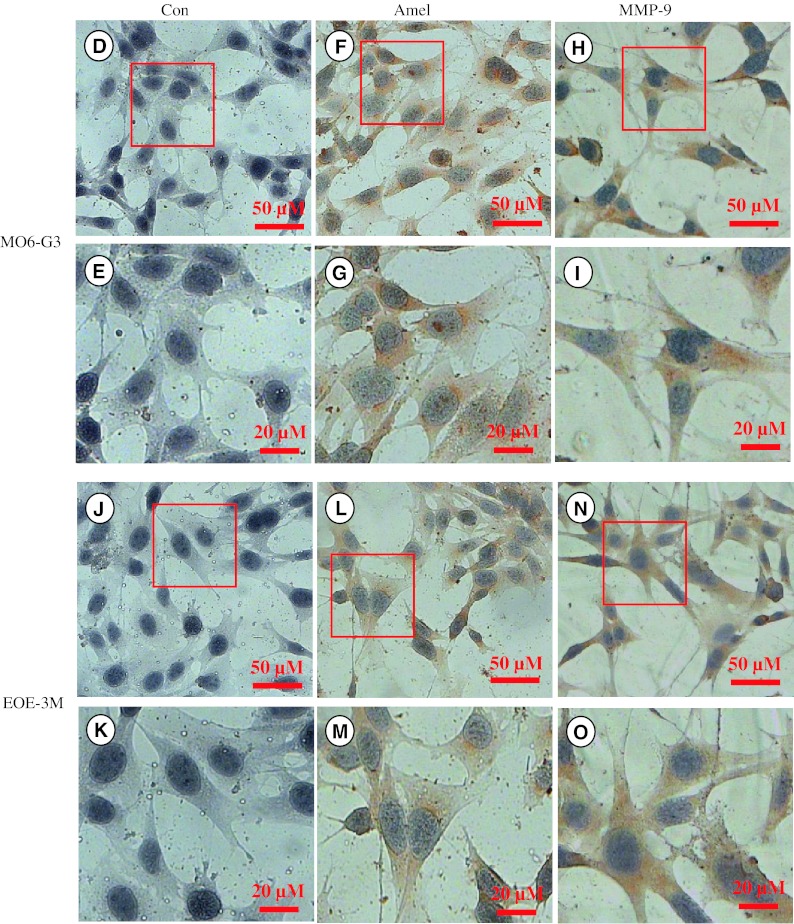
Expression of amelogenin and MMP-9 genes in mouse enamel organ epithelial and odontoblast-like cells. **a** RT-PCR analysis of mRNA expression of amelogenin and MMP-9 genes from mouse tooth tissues, EOE-3M and MO6-G3 cells. RT-PCR products were run on a 1.5 % agarose gel and stained with ethidium bromide. *M* DNA marker. *Lanes 2*, *6* are the negative control. The PCR products are 91 bp for amelogenin (*lanes 3*–*5*) and 348 bp for MMP-9 (*lanes 7*–*9*), respectively. **b** Expression of amelogenin protein in EOE-3M and MO6-G3 cells was performed by using Western blot analysis. Whole cell lysates were separated on a 10 % SDS–polyacrylamide gel and electroblotted onto Trans-Blot membranes. The blot was probed with anti-amelogenin antibody and an *arrow* indicates a detected signal. **c** Gelatin zymography was used to detect MMP-9 protein. *Lanes 1*, *2* show recombinant mouse MMP-9 protein (R&D Systems Inc.). *Lanes 3*, *4* were supernatant from EOE-3M and MO6-G3 cells without serum treatment, respectively. Recom MMP-9 indicates a recombinant mouse MMP-9 protein. **d**–**o** Expression of amelogenin and MMP-9 proteins in the EOE-3M and MO6-G3 cells was analyzed by immunostaining with either primary anti-amelogenin (**f**, **g**, **l**, **m**) or anti-MMP-9 antibody (**h**, **i**, **n**, **q**). Negative control was shown in **d**, **e**, **i** and **k**. Both amelogenin and MMP-9 signals were detected within the cytoplasm and cellular branches in EOE-3M and MO6-G3 cells

To assess amelogenin and MMP-9 expression in EOE-3M and MO6-G3 cells, RT-PCR analysis was performed using specific primers for amelogenin and MMP-9. Figure 5a showed that amelogenin and MMP-9 RNAs were identified in mouse tooth tissues, EOE-3M and MO6-G3 cells. Furthermore, their protein expression was detected by Western blot and enzymatic zymography assays, respectively (Fig. 5b, c). Immunohistochemical analysis demonstrated that those proteins were expressed within the cytoplasm and cellular branches in EOE-3M and MO6-G3 cells (Fig. 4d–o).

### MMP-9 binds to amelogenin in vitro

**Fig. 6:**
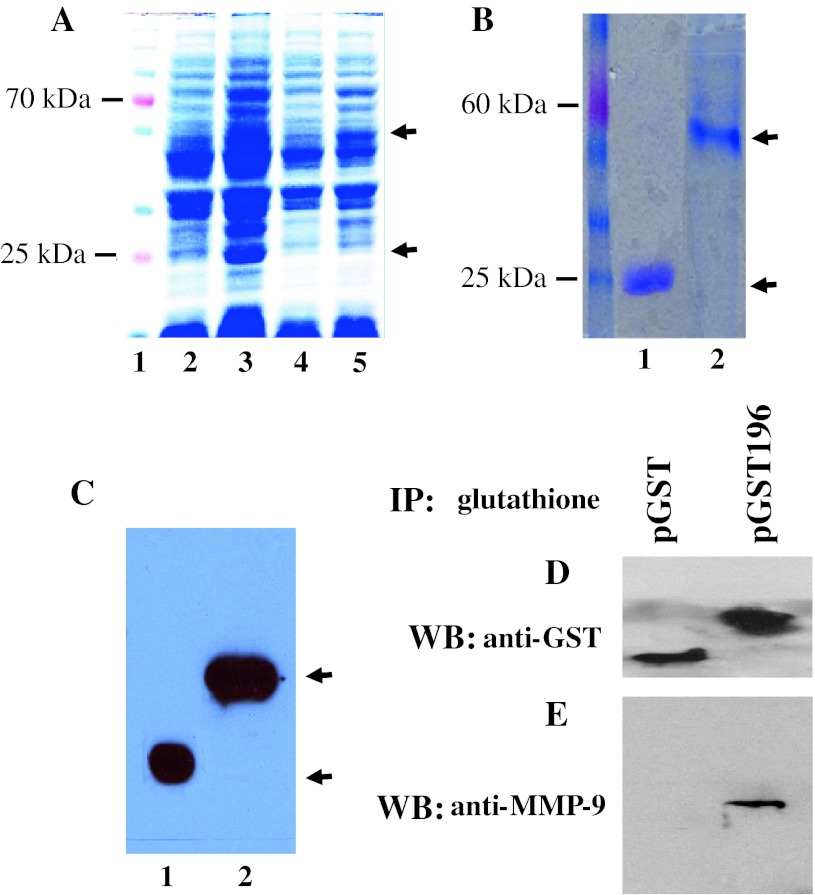
Binding of amelogenin to MMP-9 in Vitro. **a** Coomassie-stained SDS-PAGE gel for analysis of expression of untagged pET179 and pGST196 fusion proteins in DE 3 cells. *Lane 1* molecular weight marker; *lane 2* pET179 without 1 mM IPTG induction; *lane 3* pET179 with 1 mM IPTG induction; *lane 4* pGST196 without 1 mM IPTG induction; *lane 5* pGST196 with 1 mM IPTG induction. All samples were grown 3 h either with or without 1 mM IPTG induction after reaching OD600 of 0.6. *Arrows* show positions of expression of the recombinant amelogenin proteins. **b** SDS-PAGE analysis of purified pET179 (*lane 1*) and pGST196 (*lane 2*) fusion proteins from *E. coli*. extracts. **c** Expression of purified pET179 (*lane 1*) and pGST196 (*lane 2*) amelogenin proteins was confirmed by Western blotting analysis using goat polyclonal anti-amelogenin antibody (C-19, Santa Cruz Biotechnology Inc.). **d**, **e** Either 5 μg of GST-amelogenin fusion protein (pGST196) or 5 μg of GST protein alone was mixed with the recombinant mutant MMP-9 protein in binding buffer (20 mM Tris–HCl, pH 8.0, 200 mM NaCl, 1 mM EDTA) and followed by adding 20 μl of glutathione-agarose beads for further incubation. After the binding reaction, the beads were washed twice with binding buffer and once with washing buffer. Beads were boiled in 1× SDS loading dye and the samples were divided into equal aliquots, and the eluted proteins were separated by SDS-PAGE and electrotransferred to the trans-blot membranes. Western blot assay was carried out using polyclonal anti-GST antibody (Amersham Pharmacia Biotech) (**d**) and polyclonal anti-MMP-9 antibody (Santa Cruz Biotechnology Inc.) (**e**)

To determine binding of amelogenin to MMP-9, the recombinant amelogenin proteins from pET179 and pGST196 vectors were expressed in *E.coli* BL21 (DE3) cells. The crude extracts from sonicated cells were analyzed by SDS-PAGE to verify protein expression (Fig. 6a). The SDS-PAGE gel showed that the recombinant proteins, pET179 and pGST196, appeared at about 25 and 52 kDa, respectively. There was little sign of amelogenin expression in the un-induced cells. Following purification with glutathione beads of the pGST196 fusion protein, the recombinant amelogenin was resolved to near homogeneity as analyzed by SDS-PAGE gel (Fig. 6b) and further confirmed by Western blot analysis (Fig. 6c). For in vitro binding analysis, beads bearing either GST or pGST196 fusion protein were mixed with recombinant mutant MMP-9 protein. After binding and washing, bound proteins were eluted from the beads. The eluted proteins were separated by a SDS-PAGE gel and electrotransferred to a trans-blot membrane for Western blotting assay. These results showed that beads bearing GST-amelogenin protein pulled down MMP-9, whereas beads bearing GST alone failed to bind to MMP-9 protein (Fig. 6d, e). This result indicates that the amelogenin binds MMP-9 in vitro.

### Prediction of MMP-9 cleavage site(s) in amelogenin

Using computer software program, we searched for potential cleavage site(s) of MMP-9 in amelogenin across different species. The results show that the MMP-9 scissile bonds were found in amelogenin proteins across different species (Table 1).

**Table 1:** Identification of potential MMP-9 cleavage sites of amelogenin from different species

	Peptide sequences	References	Genbank accession no.
MMP-9 consensus	P X X ↓ Hy S/T	Kridel et al. 2001	
Cow	P G Y ↓ I N	Iwase et al. 2003	BAC66108
Frog	P G Y ↓ V N	Toyosawa et al. 1998	AAC78135
Guinea	P G Y ↓ I N	Cerny 1998	CAA09957
Human	P G Y ↓ I N	Salido et al. 1992	AAA51717
Mouse	P G Y ↓ I N	Snead et al. 1985	BAA06546
Pig	P G Y ↓ I N	Iwase et al. 2003	BAC66111
Rat	P G Y ↓ I N	Li et al. 1995	AAB03481

*P* proline, *Hy* hydrophobic amino acids, *S* serine, *T* threonine, *G* glycine, *Y* tyrosine, *I* isoleucine, *N* asparagine, *X* any amino acids. Scissile bonds are shown with ↓

## Discussion

Although amelogenin and MMP-9 expression during tooth development was described (Snead et al. 1988; Diekwisch et al. 1997; Sahlberg et al. 1999; Linsuwanont et al. 2002; Goldberg et al. 2003), their co-expression patterns during mouse tooth development and the interaction between the two proteins have not been investigated. In the present study, we investigated the spatial distribution of amelogenin and MMP-9 during mouse tooth development using immunohistochemistry assays. Also, we tested the interaction between amelogenin and MMP-9. Our results showed that the spatial distribution of amelogenin and MMP-9 is co-localized in ameloblasts, enamel matrix and odontoblasts during mouse tooth development although their expression levels and temporal expression patterns sometimes varied during tooth morphogenesis. Furthermore, amelogenin is able to bind MMP-9 in vitro and the MMP-9 cleavage sites exist in amelogenin proteins across different species.

Amelogenin expression was identified in the enamel organ of mouse molars as early as the embryonic day 15 (Couwenhoven and Snead 1994) and its expression was still visible in maturation stage ameloblasts at postnatal day 14 in mouse molars (Hu et al. 2001) whereas MMP-9 signal was found both in the dental epithelium and the mesenchyme at the bud stage (embryonic day 12) of developing mouse teeth (Sahlberg et al. 1999; Goldberg et al. 2003; Yoshiba et al. 2003). At the postnatal stages of tooth formation, expression of MMP-9 and other MMP family members were present in the differentiating ameloblasts (Yoshiba et al. 2003; Fanchon et al. 2004; Bourd-Boittin et al. 2005; Paiva et al. 2009). We observed co-expression of both amelogenin and MMP-9 in pre-secretory ameloblasts, secretory ameloblasts and odontoblasts as well as stratum intermedium at postnatal days 2–7 in mouse molars. Amelogenin accumulation increases with the gradient of the ameloblast differentiation and reaches an apparent plateau at the secretory stage. For the MMP-9 gene, its expression overlapped with amelogenin, in addition to the osteogenic mesenchyme and dental pulp cells. At postnatal days 5 and 7, the MMP-9 expression profile was similar to that of PN 2. Low amelogenin expression levels were present in odontoblasts and stratum intermedium cells through the postnatal stages of tooth development. This evidence was further verified in mouse enamel organ epithelial (EOE-3M) and odontoblast-like (MO6-G3) cells. Previous studies indicated that amelogenin expression was not detected in odontoblasts and other cell types (Snead et al. 1988; Inai et al. 1991, 1996; Bleicher et al. 1999; Hu et al. 2001). However, recent studies have shown that amelogenin expression is present in odontoblastic cells using in situ hybridization, immunohistochemistry and RT-PCR analyses (Oida et al. 2002; Papagerakis et al. 2003; Iacob and Veis 2006). Our results are in agreement with previous studies by other groups (Oida et al. 2002; Papagerakis et al. 2003; Iacob and Veis 2006). However, the biological roles of amelogenin in odontoblasts and stratum intermedium cells are not known. Besides co-expression of amelogenin and MMP-9 in ameloblast and odontoblasts, we further found that MMP-9 is able to interact with amelogenin, suggesting that amelogenin is a novel partner of MMP-9.

The amelogenin protein comprises about 90 % of the enamel matrix proteins and has a range of molecular masses due to alternative splicing and proteolytic cleavage. Proteolytic processing of nascent amelogenin molecules serves to generate proper amelogenin fragments as enamel development proceeds (Fincham and Moradian-Oldak 1993; Bartlett and Simmer 1999). Studies have revealed that the NH_2_-terminal and COOH-terminal domains of amelogenin proteins are essential for proper enamel formation (Gibson et al. 1991b; Le et al. 2007; Warotayanont et al. 2008; Nakayama et al. 2010; Pugach et al. 2010). In contrast, inappropriate processing of amelogenin by proteinases causes enamel defects (Caterina et al. 2002; Hart et al. 2004; Kim et al. 2005; Simmer et al. 2009).

MMP-9 is a member of the MMP family and has a broad range of substrates and mediates extracellular matrix remodeling (Kridel et al. 2001; Somerville et al. 2003; Lund et al. 2011). It is involved in normal physiological processes including bone remodeling and tooth eruption (Linsuwanont et al. 2002; Basi et al. 2011) and in pathological processes like periodontal disease (Rai et al. 2008; Silva et al. 2008), dental caries (Chaussain-Miller et al. 2006; Shimada et al. 2009) and cancer invasion (Patel et al. 2011). In MMP-9 null mice, impairment of ossification and vascularization of the skeletal growth plates was observed (Vu et al. 1998). Skeletal growth plates of MMP-9 null mice in a culture system showed a delayed release of an angiogenic activator, establishing a role for this enzyme in controlling angiogenesis. Engsig et al. (2000) demonstrated that MMP-9 is essential for the recruitment of osteoclasts into developing bone. Our study found that mice lacking MMP-9 gene exhibit abnormal tooth morphology, immature ameloblast differentiation, loss of ameloblast polarization and delayed tooth eruption as well as an increased amelogenin expression during amelogenesis compared to the wild-type mice (Yuan et al. 2009 and unpublished data).

Amelogenin has been suggested as a possible substrate for MMP-9 due to the reciprocal concentrations of amelogenin and MMP-9 observed during tooth formation (Fanchon et al. 2004; Bourd-Boittin et al. 2005). Fanchon et al. used two MMP inhibitors, Marimastat, a general MMP inhibitor, or CT_1166_, a more selective inhibitor of MMP-2 and MMP-9, and found that when mouse tooth germs were treated with either Marimastat or CT_1166_, gelatinase activity was inhibited, resulting in disturbance of murine amelogenesis. This suggests that MMP-9 is involved in amelogenin processing and enamel development. However, although MMP-9 interacts with amelogenin in vitro and there are potential MMP-9 cleavage sites in amelogenin genes across species, whether MMP-9, like MMP20 and KlK4, is capable of catalyzing amelogenin and its role during amelegenesis needs to be further investigated.
